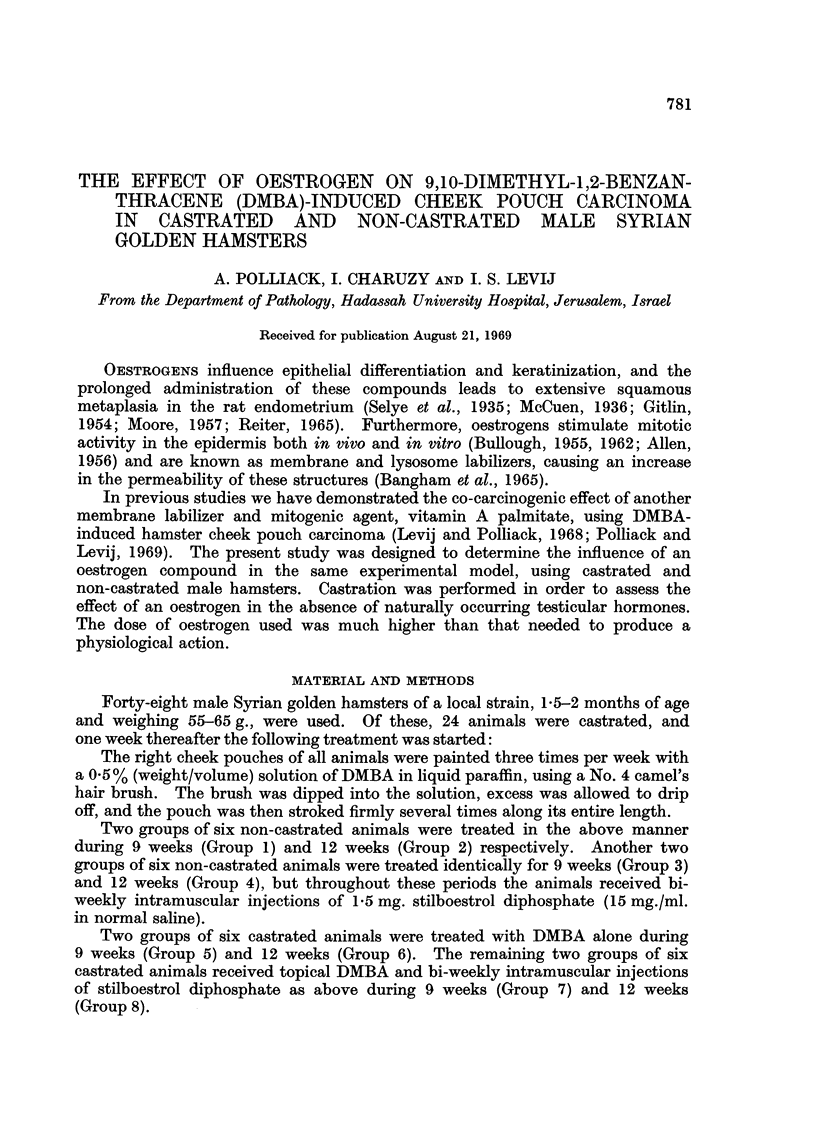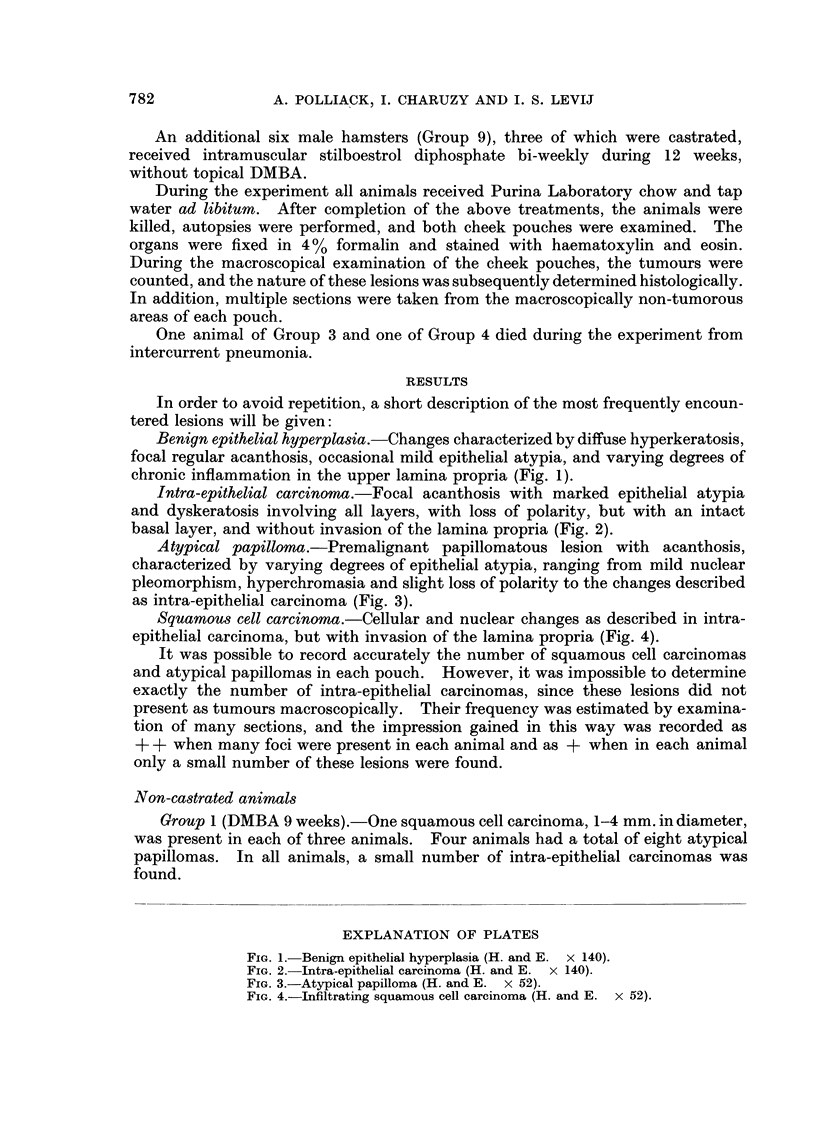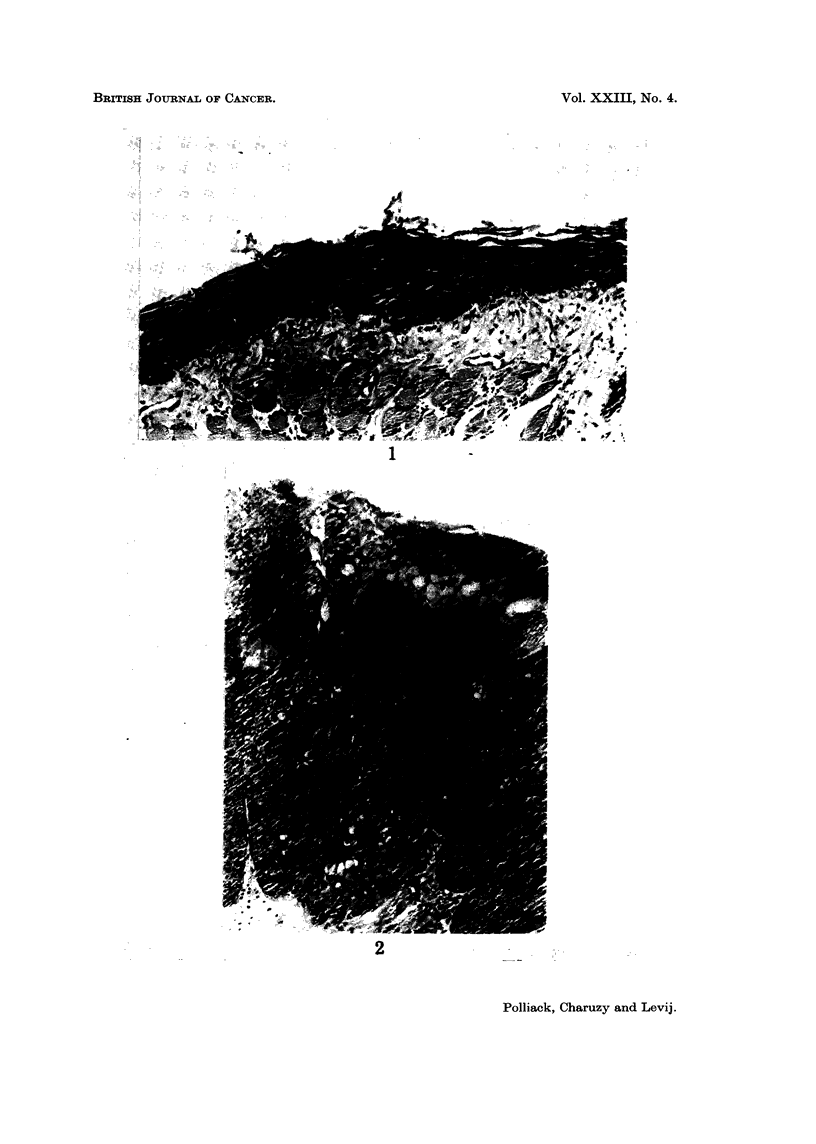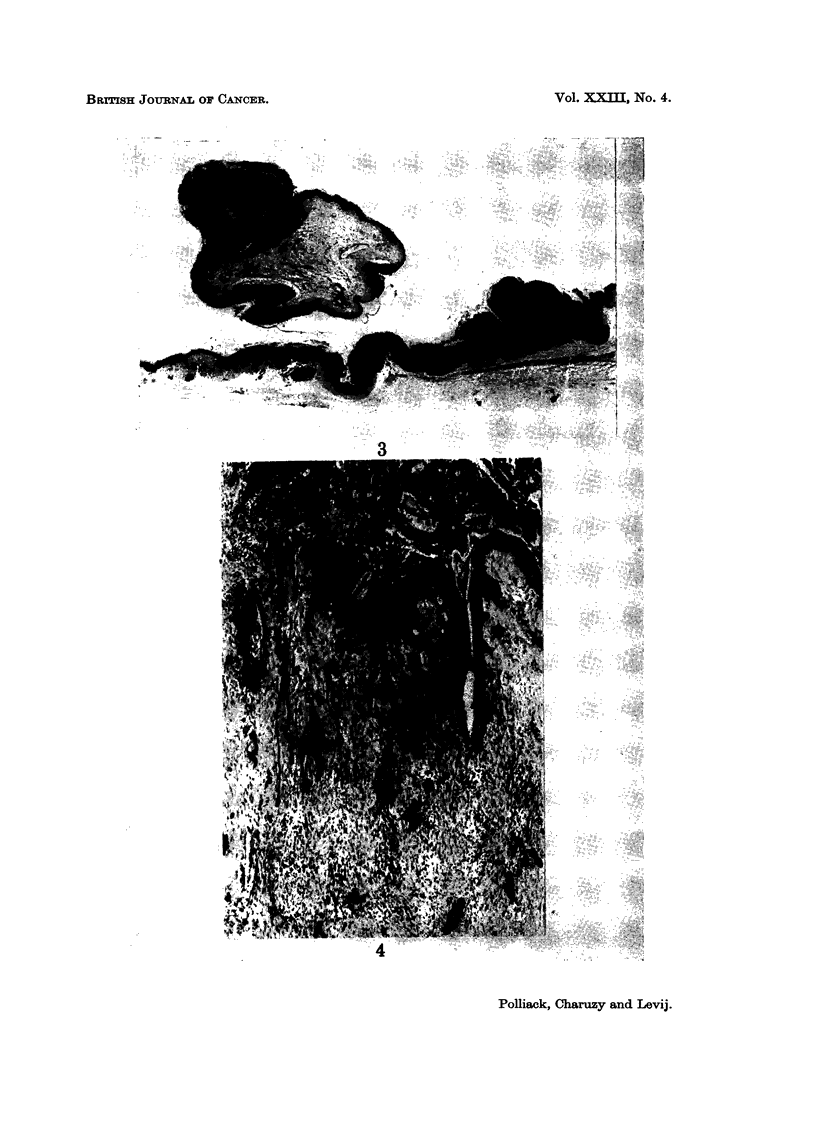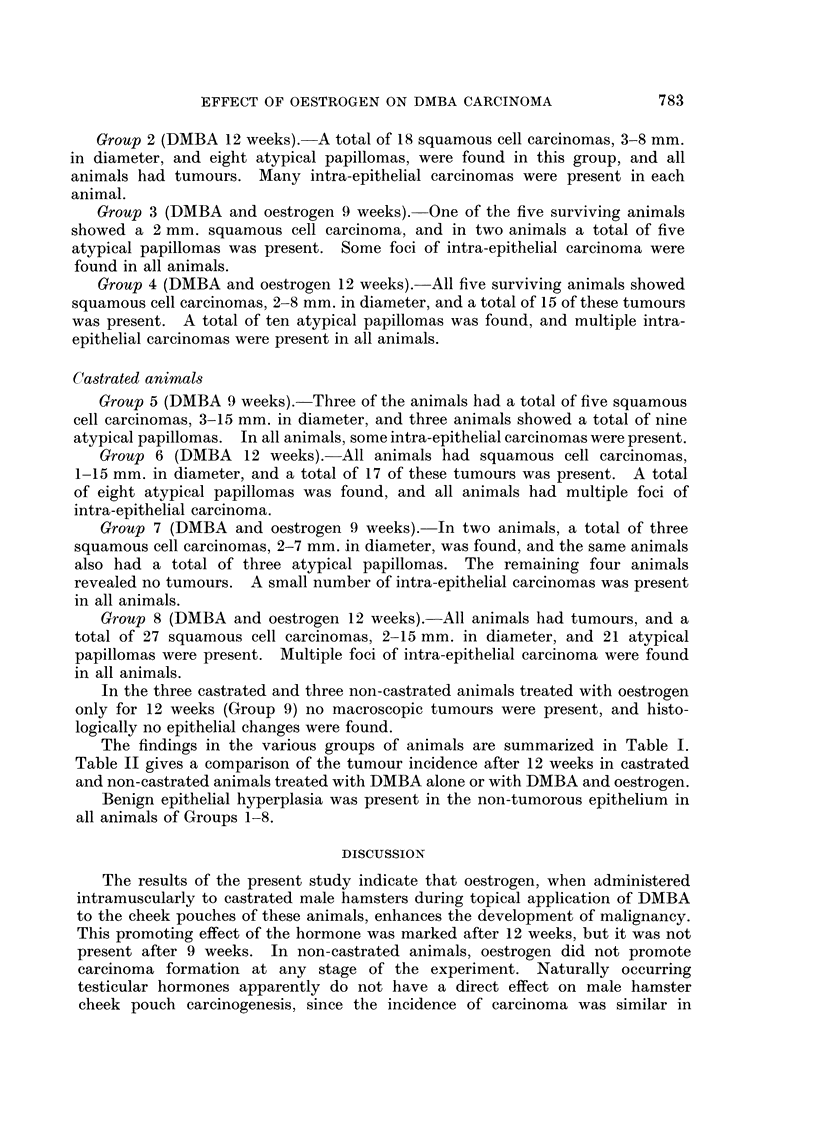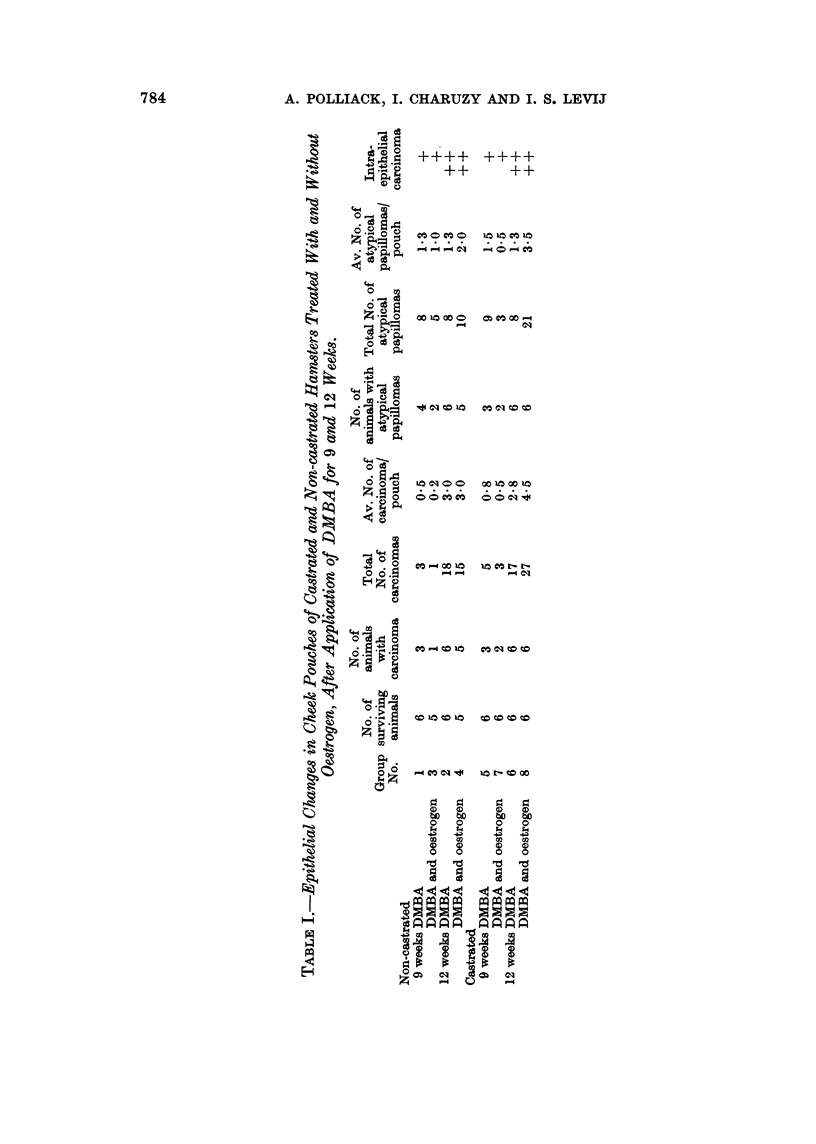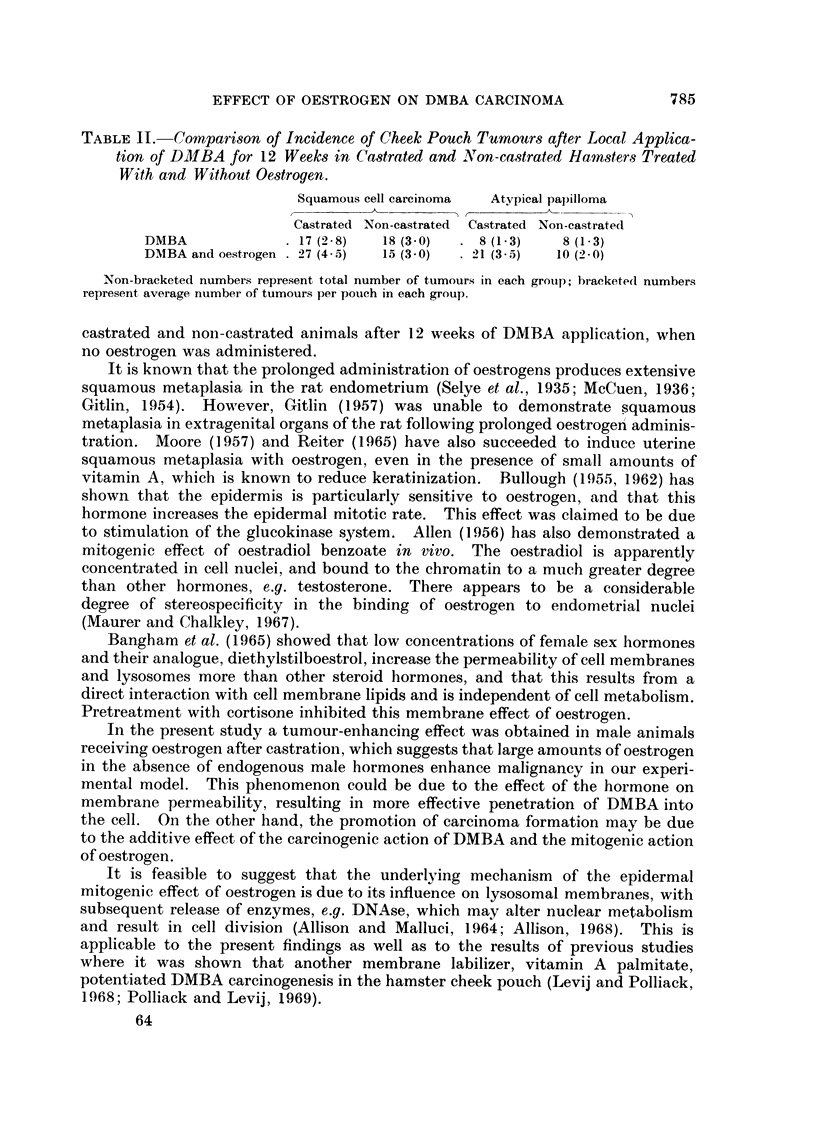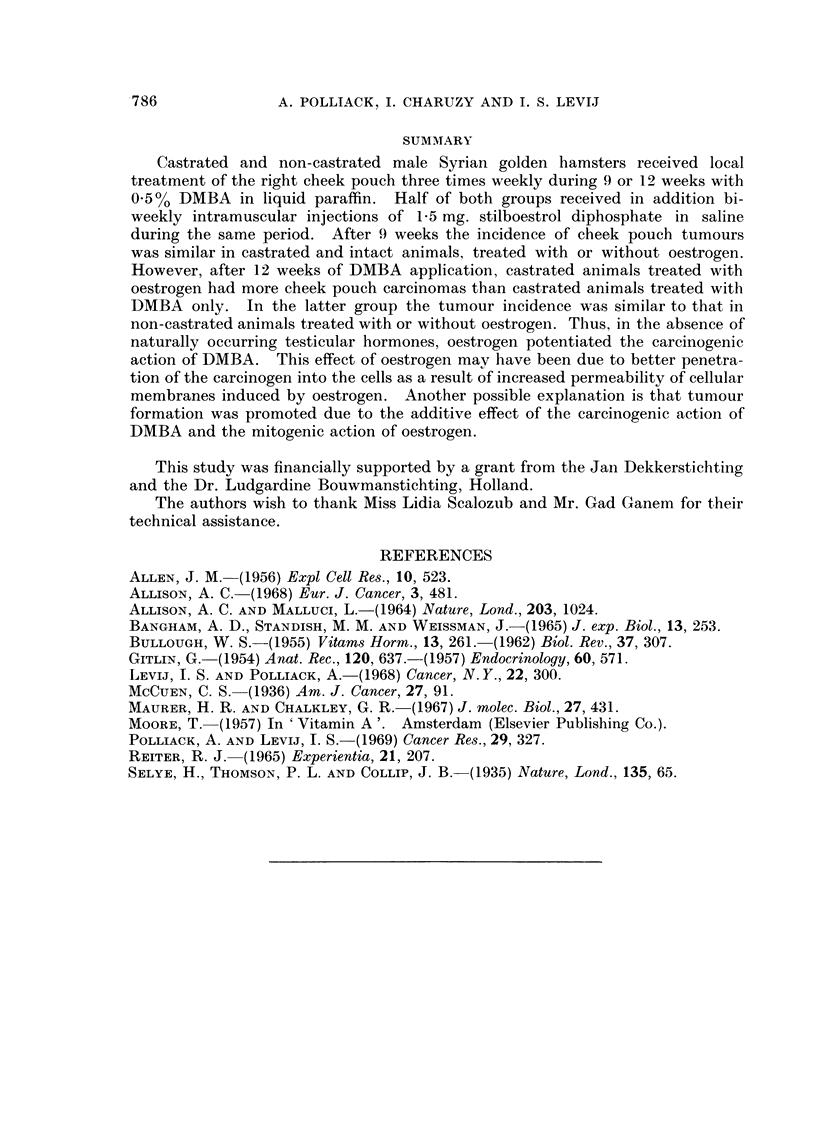# The effect of oestrogen on 9,10-dimethyl-1,2-benzanthracene (DMBA)-induced cheek pouch carcinoma in castrated and non-castrated male Syrian golden hamsters.

**DOI:** 10.1038/bjc.1969.97

**Published:** 1969-12

**Authors:** A. Polliack, I. Charuzy, I. S. Levij

## Abstract

**Images:**


### THE EFFECT OF OESTROGEN ON 9,10-DIMETHYL-1,2-BENZANTHRACENE (DMBA)-INDUCED CHEEK POUCH CARCINOMA IN CASTRATED AND NON-CASTRATED MALE SYRIAN GOLDEN HAMSTERS

A. POLLIACK, I. CHARUZY AND I. S. LEVIJ

*From the Department of Pathology, Hadassah University Hospital, Jerusalem, Israel*

Received for publication August 21, 1969

OESTROGENS influence epithelial differentiation and keratinization, and the prolonged administration of these compounds leads to extensive squamous metaplasia in the rat endometrium (Selye *et al.*, 1935; McCuen, 1936; Gitlin, 1954; Moore, 1957; Reiter, 1965). Furthermore, oestrogens stimulate mitotic activity in the epidermis both *in vivo* and *in vitro* (Bullough, 1955, 1962; Allen, 1956) and are known as membrane and lysosome labilizers, causing an increase in the permeability of these structures (Bangham *et al.*, 1965).

In previous studies we have demonstrated the co-carcinogenic effect of another membrane labilizer and mitogenic agent, vitamin A palmitate, using DMBA-induced hamster cheek pouch carcinoma (Levij and Polliack, 1968; Polliack and Levij, 1969). The present study was designed to determine the influence of an oestrogen compound in the same experimental model, using castrated and non-castrated male hamsters. Castration was performed in order to assess the effect of an oestrogen in the absence of naturally occurring testicular hormones. The dose of oestrogen used was much higher than that needed to produce a physiological action.

### MATERIAL AND METHODS

Forty-eight male Syrian golden hamsters of a local strain, 1·5–2 months of age and weighing 55–65 g., were used. Of these, 24 animals were castrated, and one week thereafter the following treatment was started:

The right cheek pouches of all animals were painted three times per week with a 0·5% (weight/volume) solution of DMBA in liquid paraffin, using a No. 4 camel's hair brush. The brush was dipped into the solution, excess was allowed to drip off, and the pouch was then stroked firmly several times along its entire length.

Two groups of six non-castrated animals were treated in the above manner during 9 weeks (Group 1) and 12 weeks (Group 2) respectively. Another two groups of six non-castrated animals were treated identically for 9 weeks (Group 3) and 12 weeks (Group 4), but throughout these periods the animals received bi-weekly intramuscular injections of 1·5 mg. stilboestrol diphosphate (15 mg./ml. in normal saline).

Two groups of six castrated animals were treated with DMBA alone during 9 weeks (Group 5) and 12 weeks (Group 6). The remaining two groups of six castrated animals received topical DMBA and bi-weekly intramuscular injections of stilboestrol diphosphate as above during 9 weeks (Group 7) and 12 weeks (Group 8).

An additional six male hamsters (Group 9), three of which were castrated, received intramuscular stilboestrol diphosphate bi-weekly during 12 weeks, without topical DMBA.

During the experiment all animals received Purina Laboratory chow and tap water *ad libitum*. After completion of the above treatments, the animals were killed, autopsies were performed, and both cheek pouches were examined. The organs were fixed in 4% formalin and stained with haematoxylin and eosin. During the macroscopical examination of the cheek pouches, the tumours were counted, and the nature of these lesions was subsequently determined histologically. In addition, multiple sections were taken from the macroscopically non-tumorous areas of each pouch.

One animal of Group 3 and one of Group 4 died during the experiment from intercurrent pneumonia.

### RESULTS

In order to avoid repetition, a short description of the most frequently encountered lesions will be given:

*Benign epithelial hyperplasia.*—Changes characterized by diffuse hyperkeratosis, focal regular acanthosis, occasional mild epithelial atypia, and varying degrees of chronic inflammation in the upper lamina propria (Fig. 1).

*Intra-epithelial carcinoma.*—Focal acanthosis with marked epithelial atypia and dyskeratosis involving all layers, with loss of polarity, but with an intact basal layer, and without invasion of the lamina propria (Fig. 2).

*Atypical papilloma.*—Premalignant papillomatous lesion with acanthosis, characterized by varying degrees of epithelial atypia, ranging from mild nuclear pleomorphism, hyperchromasia and slight loss of polarity to the changes described as intra-epithelial carcinoma (Fig. 3).

*Squamous cell carcinoma.*—Cellular and nuclear changes as described in intra-epithelial carcinoma, but with invasion of the lamina propria (Fig. 4).

It was possible to record accurately the number of squamous cell carcinomas and atypical papillomas in each pouch. However, it was impossible to determine exactly the number of intra-epithelial carcinomas, since these lesions did not present as tumours macroscopically. Their frequency was estimated by examination of many sections, and the impression gained in this way was recorded as ++ when many foci were present in each animal and as + when in each animal only a small number of these lesions were found.

### *Non-castrated animals*

*Group* 1 (DMBA 9 weeks).—One squamous cell carcinoma, 1–4 mm. in diameter, was present in each of three animals. Four animals had a total of eight atypical papillomas. In all animals, a small number of intra-epithelial carcinomas was found.

---

### EXPLANATION OF PLATES

FIG. 1.—Benign epithelial hyperplasia (H. and E. × 140).
FIG. 2.—Intra-epithelial carcinoma (H. and E. × 140).
FIG. 3.—Atypical papilloma (H. and E. × 52).
FIG. 4.—Infiltrating squamous cell carcinoma (H. and E. × 52).

1

2

Polliack, Charuzy and Levij.

3

4

Polliack, Charuzy and Levij.

*Group* 2 (DMBA 12 weeks).—A total of 18 squamous cell carcinomas, 3–8 mm. in diameter, and eight atypical papillomas, were found in this group, and all animals had tumours. Many intra-epithelial carcinomas were present in each animal.

*Group* 3 (DMBA and oestrogen 9 weeks).—One of the five surviving animals showed a 2 mm. squamous cell carcinoma, and in two animals a total of five atypical papillomas was present. Some foci of intra-epithelial carcinoma were found in all animals.

*Group* 4 (DMBA and oestrogen 12 weeks).—All five surviving animals showed squamous cell carcinomas, 2–8 mm. in diameter, and a total of 15 of these tumours was present. A total of ten atypical papillomas was found, and multiple intra-epithelial carcinomas were present in all animals.

*Castrated animals*

*Group* 5 (DMBA 9 weeks).—Three of the animals had a total of five squamous cell carcinomas, 3–15 mm. in diameter, and three animals showed a total of nine atypical papillomas. In all animals, some intra-epithelial carcinomas were present.

*Group* 6 (DMBA 12 weeks).—All animals had squamous cell carcinomas, 1–15 mm. in diameter, and a total of 17 of these tumours was present. A total of eight atypical papillomas was found, and all animals had multiple foci of intra-epithelial carcinoma.

*Group* 7 (DMBA and oestrogen 9 weeks).—In two animals, a total of three squamous cell carcinomas, 2–7 mm. in diameter, was found, and the same animals also had a total of three atypical papillomas. The remaining four animals revealed no tumours. A small number of intra-epithelial carcinomas was present in all animals.

*Group* 8 (DMBA and oestrogen 12 weeks).—All animals had tumours, and a total of 27 squamous cell carcinomas, 2–15 mm. in diameter, and 21 atypical papillomas were present. Multiple foci of intra-epithelial carcinoma were found in all animals.

In the three castrated and three non-castrated animals treated with oestrogen only for 12 weeks (Group 9) no macroscopic tumours were present, and histologically no epithelial changes were found.

The findings in the various groups of animals are summarized in Table I. Table II gives a comparison of the tumour incidence after 12 weeks in castrated and non-castrated animals treated with DMBA alone or with DMBA and oestrogen.

Benign epithelial hyperplasia was present in the non-tumorous epithelium in all animals of Groups 1–8.

### DISCUSSION

The results of the present study indicate that oestrogen, when administered intramuscularly to castrated male hamsters during topical application of DMBA to the cheek pouches of these animals, enhances the development of malignancy. This promoting effect of the hormone was marked after 12 weeks, but it was not present after 9 weeks. In non-castrated animals, oestrogen did not promote carcinoma formation at any stage of the experiment. Naturally occurring testicular hormones apparently do not have a direct effect on male hamster cheek pouch carcinogenesis, since the incidence of carcinoma was similar in

TABLE I.—*Epithelial Changes in Cheek Pouches of Castrated and Non-castrated Hamsters Treated With and Without Oestrogen, After Application of DMBA for 9 and 12 Weeks.*

| | Group No. | No. of surviving animals | No. of animals with carcinoma | Total No. of carcinomas | Av. No. of carcinoma/pouch | No. of animals with atypical papillomas | Total No. of atypical papillomas | Av. No. of atypical papillomas/pouch | Intra-epithelial carcinoma |
|---|---|---|---|---|---|---|---|---|---|
| Non-castrated | | | | | | | | | |
| 9 weeks DMBA | 1 | 6 | 3 | 3 | 0·5 | 4 | 8 | 1·3 | + |
| DMBA and oestrogen | 3 | 5 | 1 | 1 | 0·2 | 2 | 5 | 1·0 | + |
| 12 weeks DMBA | 2 | 6 | 6 | 18 | 3·0 | 6 | 8 | 1·3 | ++ |
| DMBA and oestrogen | 4 | 5 | 5 | 15 | 3·0 | 5 | 10 | 2·0 | ++ |
| Castrated | | | | | | | | | |
| 9 weeks DMBA | 5 | 6 | 3 | 5 | 0·8 | 3 | 9 | 1·5 | + |
| DMBA and oestrogen | 7 | 6 | 2 | 3 | 0·5 | 2 | 3 | 0·5 | + |
| 12 weeks DMBA | 6 | 6 | 6 | 17 | 2·8 | 6 | 8 | 1·3 | ++ |
| DMBA and oestrogen | 8 | 6 | 6 | 27 | 4·5 | 6 | 21 | 3·5 | ++ |

TABLE II.—*Comparison of Incidence of Cheek Pouch Tumours after Local Application of DMBA for* 12 *Weeks in Castrated and Non-castrated Hamsters Treated With and Without Oestrogen.*

| | Squamous cell carcinoma | | Atypical papilloma | |
|---|---|---|---|---|
| | Castrated | Non-castrated | Castrated | Non-castrated |
| DMBA | 17 (2·8) | 18 (3·0) | 8 (1·3) | 8 (1·3) |
| DMBA and oestrogen | 27 (4·5) | 15 (3·0) | 21 (3·5) | 10 (2·0) |

Non-bracketed numbers represent total number of tumours in each group; bracketed numbers represent average number of tumours per pouch in each group.

castrated and non-castrated animals after 12 weeks of DMBA application, when no oestrogen was administered.

It is known that the prolonged administration of oestrogens produces extensive squamous metaplasia in the rat endometrium (Selye *et al.*, 1935; McCuen, 1936; Gitlin, 1954). However, Gitlin (1957) was unable to demonstrate squamous metaplasia in extragenital organs of the rat following prolonged oestrogen administration. Moore (1957) and Reiter (1965) have also succeeded to induce uterine squamous metaplasia with oestrogen, even in the presence of small amounts of vitamin A, which is known to reduce keratinization. Bullough (1955, 1962) has shown that the epidermis is particularly sensitive to oestrogen, and that this hormone increases the epidermal mitotic rate. This effect was claimed to be due to stimulation of the glucokinase system. Allen (1956) has also demonstrated a mitogenic effect of oestradiol benzoate *in vivo.* The oestradiol is apparently concentrated in cell nuclei, and bound to the chromatin to a much greater degree than other hormones, *e.g.* testosterone. There appears to be a considerable degree of stereospecificity in the binding of oestrogen to endometrial nuclei (Maurer and Chalkley, 1967).

Bangham *et al.* (1965) showed that low concentrations of female sex hormones and their analogue, diethylstilboestrol, increase the permeability of cell membranes and lysosomes more than other steroid hormones, and that this results from a direct interaction with cell membrane lipids and is independent of cell metabolism. Pretreatment with cortisone inhibited this membrane effect of oestrogen.

In the present study a tumour-enhancing effect was obtained in male animals receiving oestrogen after castration, which suggests that large amounts of oestrogen in the absence of endogenous male hormones enhance malignancy in our experimental model. This phenomenon could be due to the effect of the hormone on membrane permeability, resulting in more effective penetration of DMBA into the cell. On the other hand, the promotion of carcinoma formation may be due to the additive effect of the carcinogenic action of DMBA and the mitogenic action of oestrogen.

It is feasible to suggest that the underlying mechanism of the epidermal mitogenic effect of oestrogen is due to its influence on lysosomal membranes, with subsequent release of enzymes, *e.g.* DNAse, which may alter nuclear metabolism and result in cell division (Allison and Malluci, 1964; Allison, 1968). This is applicable to the present findings as well as to the results of previous studies where it was shown that another membrane labilizer, vitamin A palmitate, potentiated DMBA carcinogenesis in the hamster cheek pouch (Levij and Polliack, 1968; Polliack and Levij, 1969).

SUMMARY

Castrated and non-castrated male Syrian golden hamsters received local treatment of the right cheek pouch three times weekly during 9 or 12 weeks with 0·5% DMBA in liquid paraffin. Half of both groups received in addition biweekly intramuscular injections of 1·5 mg. stilboestrol diphosphate in saline during the same period. After 9 weeks the incidence of cheek pouch tumours was similar in castrated and intact animals, treated with or without oestrogen. However, after 12 weeks of DMBA application, castrated animals treated with oestrogen had more cheek pouch carcinomas than castrated animals treated with DMBA only. In the latter group the tumour incidence was similar to that in non-castrated animals treated with or without oestrogen. Thus, in the absence of naturally occurring testicular hormones, oestrogen potentiated the carcinogenic action of DMBA. This effect of oestrogen may have been due to better penetration of the carcinogen into the cells as a result of increased permeability of cellular membranes induced by oestrogen. Another possible explanation is that tumour formation was promoted due to the additive effect of the carcinogenic action of DMBA and the mitogenic action of oestrogen.

This study was financially supported by a grant from the Jan Dekkerstichting and the Dr. Ludgardine Bouwmanstichting, Holland.

The authors wish to thank Miss Lidia Scalozub and Mr. Gad Ganem for their technical assistance.

REFERENCES

Allen, J. M.—(1956) *Expl Cell Res.*, **10**, 523.
Allison, A. C.—(1968) *Eur. J. Cancer*, **3**, 481.
Allison, A. C. and Malluci, L.—(1964) *Nature, Lond.*, **203**, 1024.
Bangham, A. D., Standish, M. M. and Weissman, J.—(1965) *J. exp. Biol.*, **13**, 253.
Bullough, W. S.—(1955) *Vitams Horm.*, **13**, 261.—(1962) *Biol. Rev.*, **37**, 307.
Gitlin, G.—(1954) *Anat. Rec.*, **120**, 637.—(1957) *Endocrinology*, **60**, 571.
Levij, I. S. and Polliack, A.—(1968) *Cancer, N.Y.*, **22**, 300.
McCuen, C. S.—(1936) *Am. J. Cancer*, **27**, 91.
Maurer, H. R. and Chalkley, G. R.—(1967) *J. molec. Biol.*, **27**, 431.
Moore, T.—(1957) In ‘ Vitamin A ’. Amsterdam (Elsevier Publishing Co.).
Polliack, A. and Levij, I. S.—(1969) *Cancer Res.*, **29**, 327.
Reiter, R. J.—(1965) *Experientia*, **21**, 207.
Selye, H., Thomson, P. L. and Collip, J. B.—(1935) *Nature, Lond.*, **135**, 65.